# Structural, Physicochemical, and Functional Properties of Electrolyzed Cassava Starch

**DOI:** 10.1155/2019/9290627

**Published:** 2019-04-18

**Authors:** Khanh Son Trinh, Thanh Binh Dang

**Affiliations:** HCMC University of Technology and Education, 01 Vo Van Ngan Street, Linh Chieu Ward, Thu Duc District, Ho Chi Minh City, Vietnam

## Abstract

Cassava starch was oxidized using the electrolysis system. Sodium chloride was added to this system at various concentrations from 0.5 to 5.0 % (w/v). The whiteness of modified starches proportionally increased based on the NaCl concentration and human eyes could recognize the difference of color. Under treatment, dents occurred on the surface of starch granule. Concentration of carbonyl and carboxyl groups was increased compared to native starch. Based on X-ray diffraction pattern, oxidized starch kept its A-type. Besides, the ratios of alpha-helix/amorphous regions remained indicating oxidation reaction mainly subjected on amorphous region. Intrinsic viscosity was used to indirectly calculate the average molecular weight of sample. Furthermore, results showed that average molecular weight was significantly reduced (from 2.09-fold to 13.22-fold) based on the reacting NaCl concentration. The increase of NaCl content related to the increase of retrogradation of treated starches. At various temperatures (30-95°C), swelling factor and clarity reflected negative and positive correlations to NaCl concentration.

## 1. Introduction

Cassava starch is one of major starch sources in Vietnam. However, native starch properties are not fit industrial requirements. Many methods have been used to modify natural starch to improve its functions in other industries. Oxidized starch has been widely used in various food and non-food products.

Main reagents used for starch oxidation are NaOCl, H_2_O_2_, (NH_4_)_2_SO_4_, NaBrO_3_, KMnO_4_, and O_3_. Except ozone, all others are harmful to environment. The new challenges of starch modification require a safer method for both manufacturer and environment. Electrolysis treatment, an electrochemical method, could be useful for starch oxidation [1]. This method just uses a small amount of NaCl that can easily be washed out from final product. Two main reactions occur during oxidation: firstly, the degradation of amylose and amylopectin molecules at alpha-1,4 glyosidic linkages and secondly, -OH groups at C-2, C-3, C-4, and C-6 of starch molecules being oxidized to =C=O groups and then to –COOH groups. Thus, these groups are indicators of the degree of oxidation. Theoretically, the reaction rate of starch with hypochlorite is remarkably affected by pH. The reaction rate is most rapid around pH 7 and very slow pH at 10 [2].

Under electrolysis, the extent of oxidation can be affected by many factors including NaCl concentration, input voltage, input ampere, distance of electrodes, and reaction time [3–5]. On that basis, with different electrolyte level, electrolyzed water will have different effects on starch modification. However, in this study, the current, voltage, reaction time, and distance of electrodes were fixed. Instead, this study was conducted to evaluate the effect of NaCl concentration in the electrolysis system on the structural, physicochemical, and functional properties of cassava starch.

## 2. Materials and Methods

### 2.1. Starch Modification

Cassava starch (400g) was suspended in 4.0 liters of NaCl solution (0.5-5.0%, w/v) in a cylindrical container (*ϕ*=12cm). Initial starch slurry was around 6.0. Two titanium electrodes (anode and cathode), which were coated with a mixture of RuO_2_, IrO_2_, and TiO_2_ for prevention of corrosion, were flooded in this suspension. These electrodes have a distance of 10 cm and were supplied by a 10V and 3A DC electric current. The treatment was conducted for 1 h at ambient temperature (30°C) with lightly continuous mixing by a magnetic stirrer. After treatment, the electric current was off and starch suspension was adjusted to pH 7 (by 1M HCl). Starch suspension was washed (three times) with 3-fold volume of distilled water and then centrifuged (2500×g, 10 min). The washed starch sediment was dried (40°C, 48h) to reach the final moisture content (~11%, w/w). CN0, CN0.5, CN0.75, CN1, CN2, and CN5 were treated starch in 0-5% NaCl solution.

### 2.2. Carbonyl and Carboxyl Content Measurement

Carboxyl contents were measured using methods of Chattopadhyay S.S [6]. Modified starch (2.0 g) was added to 0.1 N NaOH (25 ml). This mixture was allowed to stand for 30 min with occasional stirring. The slurry was centrifuged (2500×g, 10 min) and washed with distilled water to remove free chlorine. The starch was added to distilled water (300 ml) and was boiled (15 min) for complete gelatinization. The hot starch slurry was then adjusted to 450 ml with distilled water and titrated to pH 8.3 with standardized 0.01 N NaOH (phenolphthalein as indicator). A blank sample was also performed with unmodified starch. Carboxyl content was calculated as follows:

(1) Milliequivalents  of  acidity100g  starch=sample-blank×molequivalent/l  of  NaOH×100sample  weight  g,dry  basis

(2) Carboxyl  contentg/100g=milliequivalents  of  acidity100g  starch×0.045

Carbonyl content was measured using method of Smith R.J. [7]. Starch (2.0 g) was added to distilled water (100 ml). This mixture was completely gelatinized (95°C, 20 min) and then was cooled to 40°C and adjusted to pH 3.2 using 0.1N HCl. Hydroxylamine reagent (15 ml) was added. Reaction was performed at 40°C with slow stirring. After 4 h, sample was titrated to pH 3.2 with 0.1N HCl. Blank sample was prepared without starch. Hydroxylamine reagent was prepared by dissolving hydroxylamine hydrochloride (25 g) in 0.5 N NaOH (100 ml). The final volume was adjusted to 500 ml by distilled water. The carbonyl content was calculated as follows:

(3) Carbonyl  content  g/100g=blank-sampleml×acid  normality×0.028×100sample  weight  g,dry  basis

### 2.3. Solubility, Swelling Factor, Paste Clarity, and Syneresis Measurements

Solubility and swelling factor were measured using the method of Leach H.W. [8]. Starch (0.2 g) and distilled water (10 ml) were added to a centrifuge tube. This mixture was mixed carefully. The starch suspension was kept in a water bath (from 30 to 95°C). After 30 min, starch was cooled to room temperature and then was centrifuged (1000×g, 20 min). The supernatant was collected and dried at 105°C for 24 h.

(4) Solubility  SI,%=weight  of  dried  materialweight  of  starch×100%

(5) Swelling  factor  SP,g/g=weight  of  centrifugal  residueweight  of  starch-weight  dried  material

Paste clarity was measured by the method of Singhal and Kulkarni [9]. Starch slurry (from 0.5 to 4.0% in distilled water) was completely gelatinized (95°C, 20 min) and then was cooled to room temperature. The transmittance of each starch slurry was measured at 660 nm using a UV-Vis spectrophotometer. Distilled water was used as a blank sample.

The freeze-thaw stability of starch was estimated using its syneresis (%) following the method of Singhal and Kulkarni [9]. Starch slurry (6% in distilled water) was completely gelatinized (95°C, 20 min). This starch paste was cooled to room temperature and centrifuged (1000×g, 15 min). The percentage of water separated after each freeze-thaw cycle (21 h at -20°C and 3 h at room temperature) was calculated.

(6) $$\begin{array}{c} Syneresis\;\;\left(\;\%\;\right)=\frac{weight\;\;of\;\;separated\;\;water\;\;\left(g\right)}{weight\;\;of\;\;starch\;\;paste\;\;\left(g\right)\times 100} \end{array}$$

### 2.4. Intrinsic Viscosity and Average Molecular Weight Measurement

Intrinsic viscosity (*η*_i_, ml/g) had a relationship to the average molecular weight of starch that was dissolved in an alkaline solution. Starch was dissolved in 1M KOH to make a solution of 1.0 to 5.0 mg/ml. Kinematic viscosity (*η*, m^2^/s) was measured using an Ostwald viscometer (*ϕ*=0.3 mm, Ref. No 509 03, Germany) at 30-70°C. The relative viscosity (*η*_rel_)= *η*/*η*_o_ (*η*, *η*_o_ are kinematic viscosity of starch and 1M KOH solutions). The reduced viscosity (*η*_red_)= (*η*_rel_ -1)/c (c is the concentration of starch in KOH solution). Intrinsic viscosity (*η*_i_)=lim_*c*→0_*η*_*red*_. Based on the Staudinger-Mark-Houwink equation, the average molecular weight (M) of starch was calculated as *η*_i_=KM^*α*^ (K=1.18x10^−3^; *α*=0.89) [10–12].

### 2.5. Fourier Transform Infrared Spectroscopy Measurement

FTIR spectra were measured following the method of Kizil R. [13] at the wavenumber from 400 cm^−1^ to 4500 cm^−1^ (FTIR-8400S, Shimadzu, Japan). Starch (2.0 g) and KBr (0.2 g) were mixed well and pressed at 8.0 bar for 10 min before measurement.

### 2.6. X-Ray Diffraction Measurement

XRD was determined using a powder X-ray diffractometer (Model D5005, Bruker, Karlsruhe, Germany). The operating conditions were 40 kV and 40 mA with Cu-K*α* radiation of 0.15406 nm (Nickel filter; time constant, 4 s). Each scan was performed from 5 to 30° (2*θ*) [14]. DRC was calculated using the method of Nara [15] with peak-fitting software (Origin-version 7.5, OriginLab, Northampton, Mass., USA). DRC was the ratio of Ac/(Ac+Aa). In this, A_c_ was the area of crystalline portion and A_a_ was the area of amorphous portion.

### 2.7. Lab Color Space Measurement

Color of sample was measured using a Minolta CR-400 Chroma Colorimeter. The difference of color was calculated following a previous method [16].

### 2.8. Statistical Analysis

Experiments were conducted in triplicate, and the mean value was reported. Data were analyzed using analysis of variance (ANOVA), and the mean separations were determined by Duncan's test (p<0.05). All statistical analyses were carried out using SPSS software (Ver. 17.0, SPSS, Chicago, IL, USA).

## 3. Result and Discussion

### 3.1. Chemical Properties of Starches

**Figure 1:**
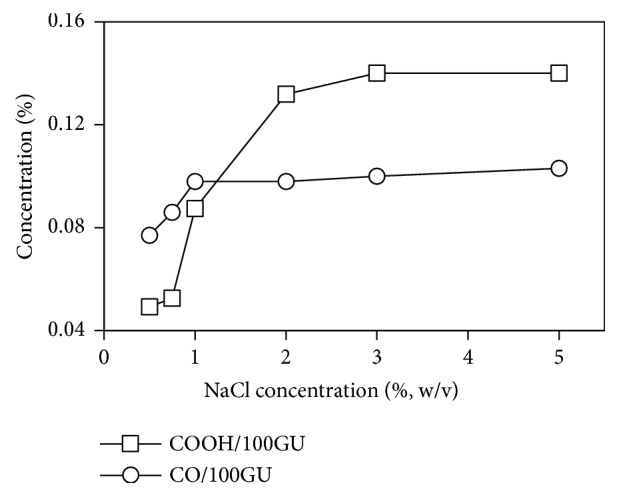
Content of carbonyl (CO/100GU) and carboxyl (COOH/100GU) of starches.

In this study (Figure 1), both carbonyl and carboxyl groups significantly increase following the increase of NaCl concentration. Theoretically, during the oxidation, the glycosidic bond cleavage caused the depolymerization of starch molecules causing the increase of carboxyl and carbonyl groups [17, 18]. Kuakpetoon D.S. and Wang Y.J. [19] suggested that the carbonyl and carboxyl contents increased with the increasing concentration of active chlorine. In this study, at low NaCl concentration (≤1%), carbonyl content is higher than carboxyl one. On the opposite, at a NaCl concentration higher than 1%, carbonyl content increased much lower than the carboxyl content. This phenomenon was explained as the hydroxyl group (-OH) on starch molecules initially were oxidized to carbonyl groups and then to carboxyl groups as the primary final product [2].

### 3.2. Morphology of Starch Granules

**Figure 2:**
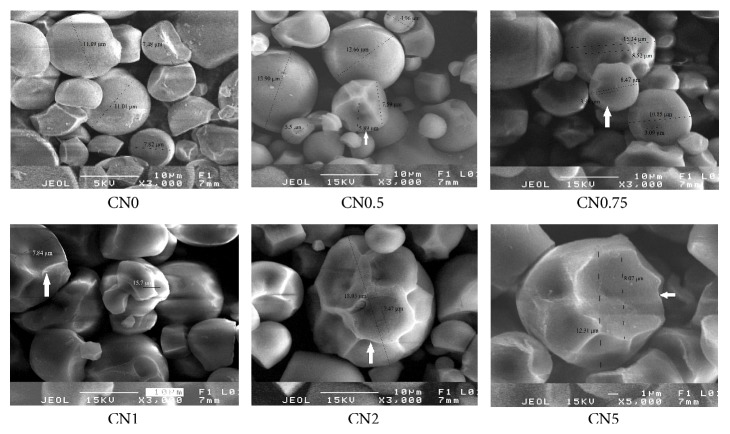
Surface of starch granules under Scanning Electron Microscopy.

Scanning electron micrographs (SEMs) of native and modified starches are shown in Figure 2. Native starch showed round shape with a truncated end on one side and a smooth surface with no evidence of any fissures or pores [20]. The truncated end of native and modified starch granules presented a rough surface. The increase of NaCl concentration resulted in rougher surface and the creation of large holes (arrows) on the surfaces of starch granules. Previous studies suggested that oxidation treatment can produce large spores in the starch granules. The amylose was distributed evenly over the entire surface and the depolymerization under treatment leading to the formation of those holes [19, 21]. Furthermore, holes observed in our study were significantly larger than those of previous ones.

### 3.3. Structural Properties of Starches

**Figure 3:**
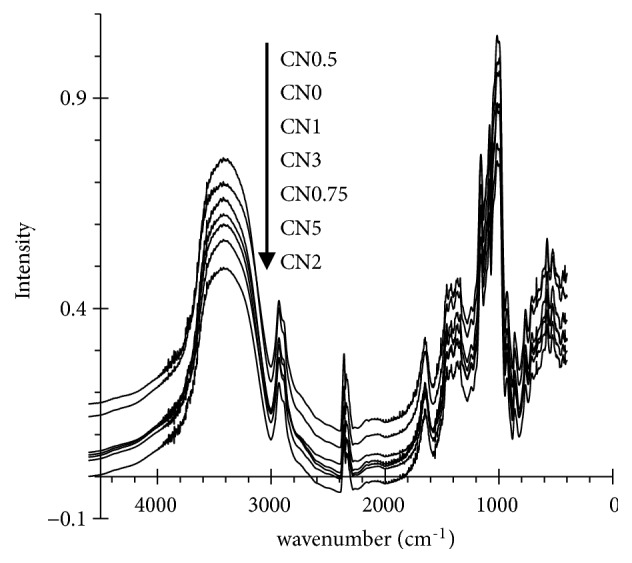
Fourier-transform infrared spectra of starches* (from the bottom to the top: CN0 to CN5)*.

Figure 3 represents the FTIR spectra of starches. These spectra patterns are similar to each other. Band absorbances in sample have been assigned and mostly matched with the vibrational modes of the chemical bonds by many previous studies. The absorbance at 3420 and 2880 cm^−1^ can be attributed to O–H and C–H bond stretching, respectively. The peaks of starch at 1650 and 1460 cm-1 were assigned as C–O–C stretching and C–H bending, respectively. The absorbances at 1150 and 1085 cm^−1^ are both assigned as the coupling of C–O, C–C, and O–H bond stretching, bending, and asymmetric stretching of the C–O–C glycosidic bridge. Absorbance at 1010 cm^−1^ is assigned to the vibration of C–O–H deformation, and absorbances at 918 and 846 cm^−1^ are assigned both for C–H bending [22]. The IR absorbance band at 1047 cm^−1^ has been considered sensitive to ordered (alpha helix) structure and the band at 1022 cm^−1^ has been associated with amorphous in starch. Thus, the ratio of intensity of 1047/1022 cm^−1^ could express the degree of order in starch [23]. In our study, this ratio slightly reduced under treatment but was similar between treated starches.

Kuakpetoon D.S. Wang Y.J. [19] suggested that amylose was more prone to depolymerization than was amylopectin at the same oxidation level. The linear chemical structure or the random arrangement of amylose made amylose more susceptible to oxidative degradation. When a large amount of oxidative agent was applied, the degradation of both amylose and amylopectin might have occurred. Intrinsic viscosity (*η*_i_) and average molecular weight (M) of starches are shown in Table 1. Obviously, electrolysis treatment resulted in significant reduction of both *η* and M. Thus, these results evidently proved the depolymerization of starch under treatment. The oxidative reaction promoted by electrolysis treatment caused partial degradation of glycosidic bonds and lowering of molecular weight that is reflected by the decrease in starch paste viscosity. Compared with hydroxyl groups, the presence of bulky carboxyl groups loosed the granular structure of starch and resulted in the decrease of the viscosity [21, 24].

**Figure 4:**
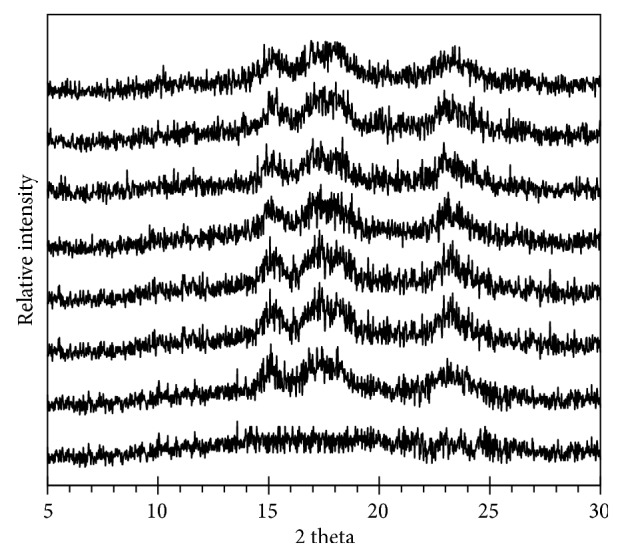
X-ray diffraction spectra of starches* (from the bottom to the top: amorphous starch, CN0 to CN5)*.

The X-ray diffraction spectra (XRD) and relative crystallinity degree (DRC) of starches are shown in Figure 4 and Table 1. Spectra of native and oxidized starches reflected A-type X-ray pattern with specific peaks at 15.1°, 17.44°, 18.12°, and 23.08° (2*θ*). Actually, the oxidation reaction caused by the electrolysis did not cause any change in X-ray pattern of starch samples [22, 25]. In our treatment, CN0.5 contained higher DRC than that of native starch. However, DRC values gradually decreased following the increase of NaCl concentration. CN2, CN3, and CN5 samples had lower DRC than that of native starch. Lúcia Helena Garrido et al. [26] and Kuakpetoon et al. [21] reported that mild treatments caused the increase of DRC, which resulted in partial reorganization of amorphous regions in starch. In contrary, harsh treatments caused the depolymerization of crystalline clusters present in amylopectin molecules leading to the disruption of DRC value. Kuakpetoon D.S. and Wang Y.J. [19] suggested that starch molecules in the amorphous lamellae were first degraded at low concentration of oxidative agent resulting in an increase in overall crystallinity. When this agent level was increased, a portion of the crystalline lamellae in sample was degraded; consequently, its crystallinity decreased. Based on the result of FITR and X-ray, we suggested that our treatment loosed crystalline structure and converted it to alpha helix regions.

### 3.4. Functional Properties of Starches

**Figure 5:**
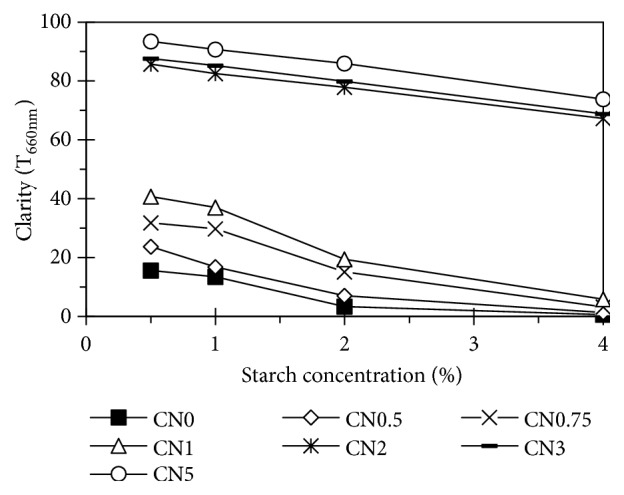
Paste clarity of starches.

Under treatment, starch samples were brighter (high L value) (Table 1). The difference of color (∆E) higher than 2 and lower than 3.5 indicates that an inexperienced person can easily detect this difference between samples.  Figure 5 shows the clarity of starch pastes. Basically, the higher the treated NaCl concentration is, the clearer the starch paste is. This result agreed with previous studies [19]. Since the bulkiness of the carboxyls and carbonyls sterically interferes with the tendency of amylose to associate and retrograde, oxidized starches produce pastes of greater clarity than that of unmodified starch [2].

**Figure 7:**
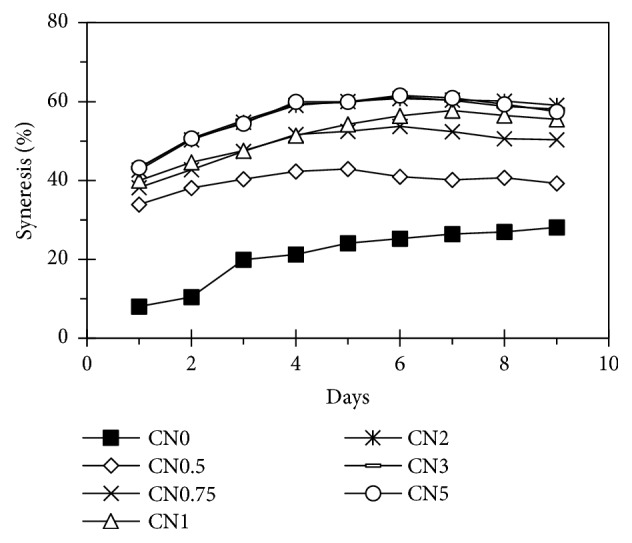
Syneresis of starches.

The syneresis tendency (retrogradation) of the starches was expressed as liquid liberated from their pastes after three freeze-thaw cycles (Figure 7). The value of treated starches was higher than that of native starches. NaCl concentration positively correlated to the syneresis of samples. Syneresis was popular for many polysaccharide gels, during which unbound water is eliminated from gel network. At early stages of gelation process, junction zones are relatively small in both number and extent. Both size and number of junction zones grew with time and so did the gel strength. Formation of precipitates and elimination of water, i.e., syneresis of gel network, were caused by further growth of junction zones. Besides, linear chain segments help to limit the size of junction zones and, hence, reduce syneresis. In addition, the loss of crystalline resulted in the increase of syneresis or water separation on starchy samples [27]. Besides, oxidation could affect the syneresis (retrogradation) of starch sample in two different ways, decreasing and increasing. The formation of carbonyl and carboxyl groups on oxidized starch molecules leads to the prevention of the chain association. Thus, sample had lower retrogradation or syneresis. On the contrary, the degradation of B_2_, B_3_ amylopectin side-chains, and amylose molecules resulted in higher retrogradation [19]. Obviously, in this study, our treatment led to the reduction of degree of crystallinity and the degradation of starch molecules. This tendency was observed in the study of Lúcia Helena Garrido et al. [26].

**Figure 8:**
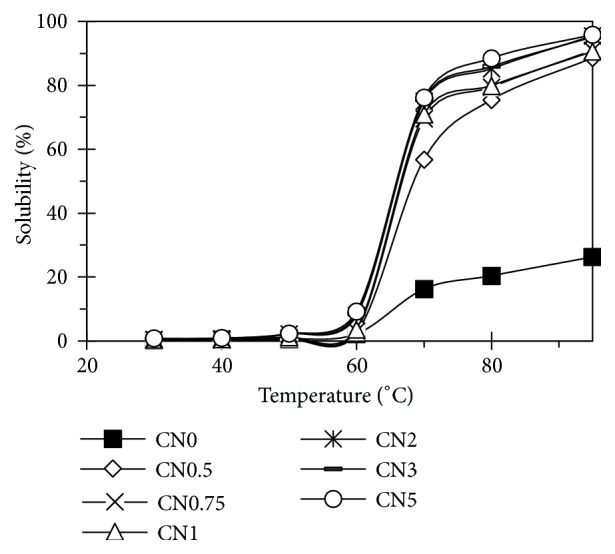
Solubility of starches.

The solubility reflected the ratio of molecules leached from the starch granule after swelling, and the swelling factor expresses the ability of starch to hydrate under specific conditions [28]. These parameters were measured at a range of 30-95°C (Figure 8). The solubility of treated starches was higher than that of raw starch. This was explained by the starch oxidation that occurs mainly in amorphous regions causing the breakdown of starch molecules to smaller ones. These formed a weakened network inside starch molecules and made them easier to absorb water leading to the increase in solubility [25, 29].

**Figure 9:**
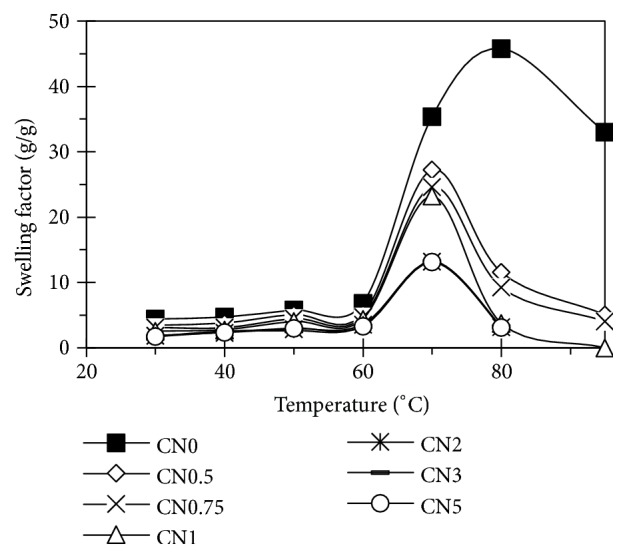
Swelling factor of starches.

At around 60°C, swelling factor of starch suddenly increased reflecting the intense hydration of sample (Figure 9). The higher the used NaCl concentration was, the lower the swelling factor and higher solubility were. Actually, the appearance of rough surface and holes of granules made them able to absorb water during heating, but they cannot retain absorbed water under centrifugation [30]. Besides, the electrolysis caused the oxidation process that may be attributed to the disintegration on the internal structure and the leaching of the amorphous region of starch granules. Thus, the starch granules more easily absorbed the water and this led to the increase in solubility and decrease in the swelling factor [24, 25].

## 4. Conclusions

Both the scission of the glycosidic linkages and oxidation of hydroxyl groups to carbonyl and carboxyl groups resulted under electrolysis treatment of granular cassava starch. Both carbonyl and carboxyl groups increased rapidly when increasing NaCl content in electrolysis reaction. These chemical changes led to the decreasing of swelling factor and paste viscosity and the increasing of paste clarity and solubility. Furthermore, oxidized starch enhanced syneresis during freeze-thaw storage. At low concentration of 0.5% NaCl, electrolysis treatment caused the increasing of crystallinity. Moreover, higher concentration of NaCl resulted in the disruption of crystal structure. However, A-type X-ray pattern remained during treatment. Besides, the treatment caused rougher surface and large holes on the surface of starch granules. Thus, with the above-described characteristics, electrolyzed starch can find many different applications in industries.

## Figures and Tables

**Figure 6:**
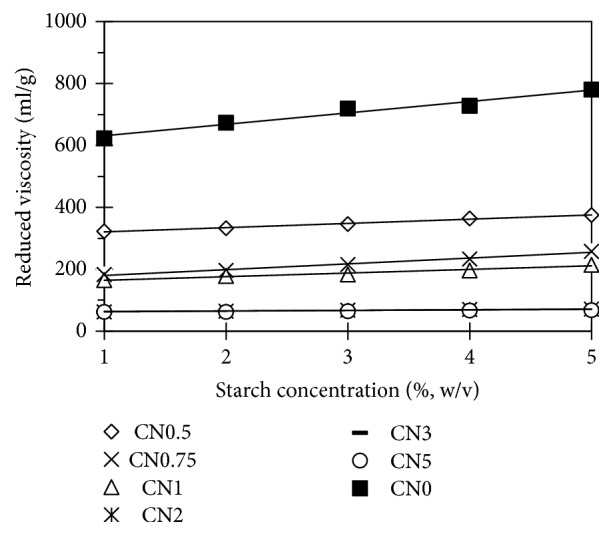
Reduced viscosity of starches.

**Table 1:** Color^*∗*^, equation of reduced viscosity, average molecular weight (M), the ratio of ordered/amorphous region (1047/1022 cm^−1^), and degree of relative crystallinity (DRC) of starches.

Sample	L^*∗*^	∆E^*∗*^	y=ax+b	R^2^	M (×10^5^ g/mol)	1047/1022 cm^−1^	DRC (%)
CN0	94.25^a^	0	y=37.1x+594.19	0.9639	25.52^d^	0.91^b^	31.9^b^
CN0.5	95.75^b^	2.23	y=13.5x+307.7	0.9911	12.18^c^	0.89^a^	40.08^d^
CN0.75	95.89^b^	2.38	y=18.7x+161.5	0.9920	5.90^b^	0.88^a^	38.8^c^
CN1	96.58^bc^	3.06	y=11.6x+152.8	0.9672	5.55^b^	0.89^a^	38.4^c^
CN2	97.91^cd^	3.80	y= 2.3x+60.3	0.9920	1.94^a^	0.88^a^	27.1^a^
CN3	97.32^d^	4.21	y=1.7x+61.5	0.9897	1.99^a^	0.89^a^	26.9^a^
CN5	97.73^d^	4.40	y=1.6x+60.2	0.9846	1.93^a^	0.89^a^	26.7^a^

L: L value in CIE Lab color space of starches; ∆E: the difference in color of starches; y=ax+b: regression from Figure 6. In this equation, b is intrinsic viscosity (*η*_i_, ml/g); R^2^: R square value of the equation.

## Data Availability

(1) The [FIGURES] and [TABLE] data used to support the findings of this study are included within the Supplementary Materials file. (2) The [Figure 3] original data used to support the findings of this study are available from the corresponding author upon request. (3) The [CITATIONS] data used to support the findings of this study are included within the article, which have been cited and listed in References.

## Supplementary Materials

Supplementary Materials A* Supplementary Materials* file (.xlsx) supporting the findings of this study is included.
